# Associations between protein to non-protein ratio and intakes of other dietary components in a cohort aged 65–75 years: the Nutrition for Healthy Living Study

**DOI:** 10.1017/S1368980023001726

**Published:** 2023-12

**Authors:** Rebecca Luong, Rosilene V Ribeiro, Vasant Hirani, Stephen J Simpson, David G Le Couteur, David Raubenheimer, Alison K Gosby

**Affiliations:** 1 Charles Perkins Centre, The University of Sydney, Camperdown, NSW, Australia; 2 Nutrition and Dietetics Group, Sydney Nursing School, Faculty of Medicine and Health, The University of Sydney, Camperdown, NSW, Australia; 3 ARC Centre of Excellence in Population Ageing Research (CEPAR), The University of Sydney, Camperdown, NSW, Australia; 4 School of Life and Environmental Sciences, The University of Sydney, Camperdown, NSW, Australia; 5 Centre for Education and Research on Ageing, Concord Hospital, The University of Sydney, Camperdown, NSW, Australia; 6 ANZAC Research Institute, The University of Sydney, Concord Hospital, Concord, NSW, Australia; 7 Boden Institute of Obesity, Nutrition, Exercise and Eating Disorders, The University of Sydney, Camperdown, NSW, Australia

**Keywords:** Macronutrient intake, Dietary proteins, Nutritional requirements, Nutrients, Aged

## Abstract

**Objective:**

Diets with a low proportion of energy from protein have shown to cause overconsumption of non-protein energy, known as Protein Leverage. Older adults are susceptible to nutritional inadequacy. The aim was to investigate associations between protein to non-protein ratio (P:NP) and intakes of dietary components and assess the nutritional adequacy of individuals aged 65–75 years from the Nutrition for Healthy Living (NHL) Study.

**Design:**

Cross-sectional. Nutritional intakes from seven-day weighed food records were compared with the Nutrient Reference Values for Australia and New Zealand, Australian Guide to Healthy Eating, Australian Dietary Guidelines and World Health Organisation Free Sugar Guidelines. Associations between P:NP and intakes of dietary components were assessed through linear regression analyses.

**Setting:**

NHL Study.

**Participants:**

113 participants.

**Results:**

Eighty-eight (59 female and 29 male) with plausible dietary data had a median (interquartile range) age of 69 years (67–71), high education level (86 %) and sources of income apart from the age pension (81 %). Substantial proportions had intakes below recommendations for dairy and alternatives (89 %), wholegrain (89 %) and simultaneously exceeded recommendations for discretionary foods (100 %) and saturated fat (92 %). In adjusted analyses, P:NP (per 1 % increment) was associated with lower intakes of energy, saturated fat, free sugar and discretionary foods and higher intakes of vitamin B_12_, Zn, meat and alternatives, red meat, poultry and wholegrain % (all *P* < 0·05).

**Conclusions:**

Higher P:NP was associated with lower intakes of energy, saturated fat, free sugar and discretionary. Our study revealed substantial nutritional inadequacy in this group of higher socio-economic individuals aged 65–75 years.

The phenomenon of Protein Leverage has been demonstrated in previous research: strong regulation of protein intake causes overconsumption of non-protein energy in diets with a low proportion of energy from protein^(1–3)^. A systematic review and meta-analysis of randomised controlled trials showed that when compared with carbohydrate and/or fat, protein acutely decreased the appetite-stimulating hormone ghrelin and increased the satiety hormones cholecystokinin and glucagon-like peptide-1^(4)^. Higher protein to non-protein ratios (P:NP) have been negatively associated with intakes of ultra-processed foods in adults and children^(5)^. Higher P:NP have also been positively associated with intakes of dairy and alternatives, meat and alternatives and nutrients found in those food groups including Ca and Zn and negatively associated with intakes of energy dense, nutrient poor discretionary foods in pregnant women^(6)^. However, the associations between P:NP and intakes of other dietary components in older adults who are susceptible to nutritional inadequacy have not been explored^(7)^.

Nutritional inadequacy is a leading modifiable risk factor for morbidity, disability and mortality^(7–9)^. Nutritional adequacy is defined by nutritional intake that meets the dietary requirements for the prevention of deficiency and chronic diseases^(10)^. Therefore, nutritional inadequacy encompasses dietary intake below, exceeding and/or outside of recommendations. Food groups, food subgroups and nutrients of concern with ageing include those at risk of deficiency, associated with reduced risks of chronic disease and associated with increased risks of chronic disease^(7,11–13)^. Thus, it is important to investigate whether dietary P:NP intakes are associated with intakes of other dietary components including food groups, food subgroups and nutrients in older adults, particularly in the transitional period of early old age between 65 and 75 years, where medical, physiological and social changes may influence diet^(7)^.

The current study aims to explore the cross-sectional associations between dietary P:NP and intakes of other dietary components and assess the nutritional adequacy of the habitual diet (i.e. pre-intervention) of individuals aged 65–75 years from The Nutrition for Healthy Living (NHL) study.

## Methods

### Study design

The NHL study completed between April 2017 and June 2018 was designed to investigate the effects of randomised meal delivery diet interventions on health outcomes in generally healthy individuals aged 65–75 years living in Sydney, Australia^(14)^. The NHL study protocol has been described in detail elsewhere^(14)^. The NHL study is a single-blinded parallel randomised meal delivery diet intervention that involved 113 participants. Volunteers were recruited through paper media, electronic media, radio, television and social media advertisements. Participants were eligible to attend a screening interview if they were aged 65–75 years, both male or female (post-menopausal) volunteers, with a BMI between 20 and 35 kg/m^2^, individuals who were generally well and not taking medications known to affect weight or energy expenditure. Individuals diagnosed with type 1 diabetes mellitus, insulin-dependent diabetes mellitus, renal disease, liver disease, cancer or active neoplasms and hyperthyroidism (unless treated or under control), who experienced unintentional weight loss of > 10 % body weight over the past 5 years, who were current smokers, alcohol consumers of > 3 standard drinks per day, vegetarians, had food allergies and/or intolerances and had contraindications by treating doctor to changes in diet were excluded. Data were collected between April 2017 and June 2018. The NHL study received ethics approval from Sydney Local Health District Ethics Review Committee (Royal Prince Alfred Hospital Zone). The trial was registered with the Australian and New Zealand Clinical Trials Registry (ACTRN12616001606471) on 21 November 2016.

### Dietary data

Of the 113 participants, 107 completed a pre-intervention baseline dietary assessment involving a seven-day weighed food record whom were included in the present study for cross-sectional analyses. Participants were asked to include as much detail as possible such as brand, preparation technique, leftovers, recipes and foods consumed outside of the home. An electronic scale (Ikea ORDNING scale) was provided in addition to photographic and written instructions. The recording procedure was demonstrated by the study coordinator.

Habitual nutritional intakes from baseline food records were converted to nutrient, food group and food subgroup intakes using FoodWorks 8 Professional for Windows (Xyris Software (Australia) Pty Ltd) with the Australian Food, Supplement and Nutrient Database 2013 (AUSNUT 2013)^(15)^. Dietary P:NP was calculated on the basis of protein as a percentage of energy (%E), carbohydrate %E and fat %E summed to 100 % of energy intake. Nutrient intakes were compared with the nutrient reference values for Australia and New Zealand^(16)^ according to age and sex. Estimated average requirement and adequate intake described amounts of nutrients required for adequate physiological function and prevention of deficiency diseases, whilst the suggested dietary target and acceptable macronutrient distribution range are related to chronic disease prevention^(16)^. Consumption of alcohol was compared with the no more than 10 standard drinks a week recommendation of the Australian Guidelines to Reduce Health Risks from Drinking Alcohol^(17)^. As free sugar (added sugar plus sugars in honey and fruit juice)^(18)^ was not available in FoodWorks 8, added sugar intake was compared with the recommendation of the WHO guidelines^(19)^.

Food group and food subgroup intakes were compared with the Australian Guide to Healthy Eating according to the age and sex^(20)^. Consumption of wholegrain as a percentage (%) of total grain, red meat and seafood intake were compared with the recommendations of the Australian Dietary Guidelines^(12)^. ‘Seafood’ and ‘fish’ are used interchangeably according to the Joint Food and Agriculture Organisation/WHO Expert Consultation on the Risks and Benefits of Fish Consumption^(21)^. Due to the different classification of sugar, solid fat and alcohol as food groups in FoodWorks, a serve of discretionary food was defined as 4·2 g sugar (1 teaspoon), 4·8 g solid fat equivalents (1 teaspoon) or 10 g alcohol (1 standard drink)^(22–24)^.

### Ancillary data

Ancillary data were collected through self-reported questionnaires and physical assessment^(14)^. Weight was measured to the nearest 0·01 kg using an electronic scale and height was measured using a wall mounted stadiometer, which were used to calculate the BMI.

Physical activity was assessed through the use of the Physical Activity Scale for the Elderly^(25)^. ‘High’ and ‘low’ education levels were defined as with and without post-school qualifications, respectively. ‘Age Pension only’ referred to those who only received the Age Pension, whilst ‘other’ referred to those with other sources of income apart from the Age Pension including veteran pension, repatriation pension, superannuation, private income, business ownership, farm ownership, business partnership, wage, salary and/or other. ‘Married’ referred to those who were married, living with partner or in a de facto relationship, whilst ‘not married’ referred to those who never married, were divorced, separated, widowed or other. ‘Outright owner’ indicated the individual owned their own home, whilst ‘other’ meant they were paying off their home, paying rent to government for public housing, paying rent or board to a private landlord, or leasing and purchasing or other financial plan in a retirement village. ‘Australia/New Zealand’ indicated they were born in Australia or New Zealand and ‘other’ meant they were born in a country other than Australia or New Zealand. ‘Ex-smokers’ smoked > 100 cigarettes (5 packets) in their entire life, whilst those who ‘never smoked’ smoked < 100 cigarettes in their entire life. Self-rated health categories included ‘excellent’, ‘good’, ‘fair’, ‘poor’ and ‘very poor’^(26)^. Living location was categorised as ‘metropolitan’ or ‘rural’ based on the reported postcode of residence^(27)^. For meal preparation, ‘yes’ indicated participants can plan or cook full meals for themselves without help, and ‘no’ indicated participants can prepare some things but were unable to cook full meals for themselves and required some help.

### Right-angled mixture triangle plot

The Nutritional Geometry Framework is an approach adapted from ecological studies to human nutrition that allows for the examination of the interacting effects of dietary components as opposed to single nutrient analysis^(28,29)^. In proportion-based nutritional geometry, three component mixtures such as protein, carbohydrate and fat can be represented in a two-dimensional model^(28,29)^. The right-angled mixture triangle has been used to plot the macronutrient composition of foods, meals, various animal and human diets^(28,29)^. The right-angled mixture triangle was plotted, on the basis of protein as a percentage of energy (%E), carbohydrate %E and fat %E summed to 100 % of energy intake.

### Statistical analysis

Normality tests (histogram, Q-Q plot and Shapiro–Wilk test) found that most data had a skewed distribution. Participant characteristics between those who reported and did not report habitual dietary data and across dietary P:NP tertiles were compared through *χ* ^2^, Fisher’s exact and median tests with Bonferroni correction for multiple tests where applicable.

Median (interquartile range) intakes of energy, nutrients, food groups and food subgroups were reported. Proportions of nutritional inadequacy for each nutrient, food group or food subgroup were determined by the number of participants who did not meet the recommendation divided by the total number of participants.

The associations between dietary P:NP and intakes of dietary components were evaluated through linear regression. Dietary P:NP was analysed as both continuous and categorical variables (i.e. categorised into tertiles with the lowest as the reference). Results are presented as *β* coefficients with 95 % CI. Models were adjusted for the following sociodemographic, lifestyle and health covariates: age, sex, BMI, country of birth, marital status, education level, source of income, housing arrangement, living location, smoking status, PASE, ability to prepare own meals and self-rated health. Collinearity diagnostics was conducted and there was no collinearity in the models with variance inflation factors < 2·5. *P* < 0·05 was considered statistically significant.

Data was analysed using Statistical Packages for Social Sciences (SPSS) version 25.0 software^(30)^. Graphics were performed using R software^(31)^.

### Misreporting sensitivity analysis

Misreporting of energy intake was identified using the Goldberg cut-off method for seven-day dietary data^(32)^. The BMR for each individual was calculated using the Schofield equation based on sex, age and weight at baseline^(33)^. The appropriate values for coefficients of variation and physical activity level (PAL), according to age and sex, were used to calculate the energy intake to BMR ratio (EI:BMR) cut-offs^(32)^. Each individual’s EI:BMR was compared with the EI:BMR 95 % CI cut-offs for plausible reporters: 1·07–2·45 for females aged 65–74 years (PAL 1·62), 1·01–2·17 for females aged ≥ 75 years (PAL 1·48), 1·03–2·51 for males aged 65–74 years (PAL 1·61) and 1·02–2·34 for males aged ≥ 75 years (PAL 1·54). Non-plausible reporters had EI:BMR below and above the cut-offs. To assess bias introduced by misreporting in dietary assessment, sensitivity analyses including and excluding non-plausible reporters were conducted.

Results on associations of dietary P:NP as tertiles and continuous variables with intakes of other dietary components were different when non-plausible reporters were excluded. As such, all data are presented with non-plausible reporters excluded. Participant characteristics between those with plausible and non-plausible dietary data were compared through *χ* ^2^, Fisher’s exact and median tests.

## Results

Of the 113 participants, 88 were included in the analyses (see online Supplementary Fig. S1, Online Resource). There were no differences in participant characteristics between individuals who reported dietary data (*n* 107) and those who did not report dietary data (*n* 6) (*P* > 0·05) (see online Supplementary Table S2, Online Resource). There were no differences in participant characteristics between individuals with plausible (*n* 88) and non-plausible dietary data (*n* 19) (*P* > 0·05), except that non-plausible reporters who all underreported their dietary intake had a higher BMI (*P* = 0·039) (see online Supplementary Table S3, Online Resource). Participant characteristics of individuals with plausible dietary data are reported in Table 1. Participants (59 female and 29 male) had a median (interquartile range) age of 69 years (67–71), BMI of 27·3 kg/m^2^ (25·0–29·5), high education level (86 %), other sources of income apart from the Age Pension (81 %), were outright owners of their home (77 %) and rated their self-reported health as excellent or good (82 %). There were no differences in participant characteristics between the dietary P:NP tertiles (*P* > 0·05).

**Table 1:** Participant characteristics (percentages and number of participants; median and interquartile range) across dietary protein to non-protein ratio tertiles

Characteristic	All *n* 88		Low P:NP 0·19 (0·18,0·21) *n* 29		Medium P:NP 0·24 (0·23,0·25) *n* 30		High P:NP 0·29 (0·28,0·33) *n* 29		*P* *
	*n*	%	*n*	%	*n*	%	*n*	%	
Sex									0·22
Female	67	59	55	16	70	21	76	22	
Male	33	29	45	13	30	9	24	7	
	Median	IQR	Median	IQR	Median	IQR	Median	IQR	
Age (years)	69	67–71	70	66–72	69	67–70	69	67–72	0·14
Weight (kg)	72·9	66·0–82·9	72·5	63·3–82·9	71·0	64·9–81·6	75·8	68·8–83·6	0·79
BMI (kg/m^2^)	27·3	25·0–29·5	25·9	22·6–27·7	26·8	24·3–28·5	27·6	25·9–30·1	0·19
PASE (*n* 82)	128·4	95·4–160·5	136·3	78·6–173·1	114·6	85·5–147·3	140·7	101·1–170·3	0·11
	*n*	%	*n*	%	*n*	%	*n*	%	
Education level									0·54
High	86	76	90	26	90	27	79	23	
Low	14	12	10	3	10	3	21	6	
Source of income (*n* 82)									0·15
Age pension only	19	15	18	5	29	8	8	2	
Other	81	67	82	23	71	20	92	24	
Marital status									0·19
Married	55	48	66	19	57	17	41	12	
Not married	46	40	35	10	43	13	59	17	
Housing arrangements (*n* 86)									0·85
Outright owner	77	66	76	22	79	23	72	21	
Other	23	20	21	6	20	6	28	8	
Country of birth									0·32
Australia/New Zealand	63	55	72	21	53	16	62	18	
Other	38	33	28	8	47	14	38	11	
Smoking status (*n* 87)									0·79
Ex-smokers	37	32	32	9	37	11	41	12	
Never smoked	63	55	68	19	63	19	59	17	
Self-rated health (*n* 85)									0·73
Excellent/good	82	70	86	25	79	22	100	27	
Fair/poor/very poor	18	15	14	4	21	6	0	0	
Living location									0·47
Metropolitan	86	76	83	24	83	25	93	27	
Rural	14	12	17	5	17	5	7	2	
Ability to prepare own meals (*n* 85)									0·33
Yes	98	83	93	27	100	29	100	27	
No	2	2	7	2	0	0	0	0	

P:NP, protein to non-protein; PASE, Physical Activity Scale for the Elderly.

* *P* values were obtained using the median tests with Bonferroni correction for multiple tests, *χ* ^2^ and Fisher’s exact tests to compare all tertile groups for differences in participant characteristics.

### Nutritional adequacy

**Fig. 1:**
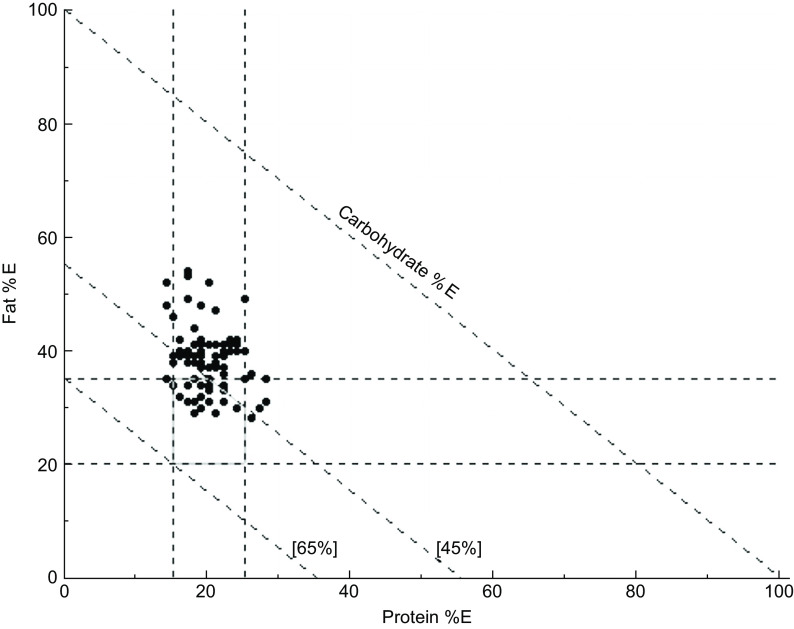
Right-angled mixture triangle (RMT) plot on baseline dietary macronutrient composition data of individuals (*n* 88) and the integrated region of the acceptable macronutrient distribution range (AMDR) from the nutrient reference values for Australian and New Zealand for fat (20–35 %), protein (15–25 %) and carbohydrate (45–65 %) is presented. Fat as a percentage of energy (%E) and protein %E increase along their respective axes and carbohydrate %E decreases with distance from the origin

Table 2 reports the intake of energy, nutrients, food groups and food subgroups and proportions of nutritional inadequacy of all participants and by tertiles of the dietary P:NP. The median intake of protein %E, carbohydrate %E and total fat %E for all participants was 17·9 %, 39·6 % and 35·9 % respectively. A small proportion of participants (19 %) consumed protein %E intakes outside of the recommendations, whereas the majority (85 %) had intakes below the recommendations for carbohydrate %E and more than half (55 %) exceeded the recommendations for total fat %E. Figure 1 plots the macronutrient composition of the habitual diet from the three components of all participants in a right-angled mixture triangle and the integrated region of the acceptable macronutrient distribution range recommendations. The range along the protein %E axis was less than along the axes for carbohydrate %E and fat %E intakes, indicating a narrower range of intakes for protein %E in comparison with carbohydrate %E and fat %E.

**Table 2:** Median daily intakes of energy, nutrients, food groups and food subgroups, recommended intake according to age and sex and the proportions of nutritional inadequacy of all participants and across dietary protein to non-protein ratio tertiles

		All *n* 88			Low P:NP 0·19 (0·18, 0·21) *n* 29			Medium P:NP 0·24 (0·23, 0·25) *n* 30			High P:NP 0·29 (0·28, 0·33) *n* 29		
Dietary component	Recommended intake	Median	IQR	*n* (%)	Median	IQR	*n* (%)	Median	IQR	*n* (%)	Median	IQR	*n* (%)
Energy (kJ)	–	7893·3	7139·9–9494·4	–	9100·8	7875·5–10 224·6	–	7541·4	6918·0–8525·7	–	7785·6	6988·1–8922·6	–
Nutrients													
Protein (g/kg BW)-EAR	F: 51–70 yo: 0·60 71+: 0·75 M: 51–70 yo: 0·68 71 yo+: 0·86	1·19	1·02–1·33	1	1·07	0·92–1·27	3	1·15	1·04–1·28	0	1·26	1·13–1·50	0
Protein (g)	–	84·3	76·2–97·6	–	78·0	72·0–89·5	–	79·5	75·5–93·9	–	95·7	83·1–114·2	–
Protein (%E)-AMDR	15–25	17·9	16·1–20·4	19	15·2	14·4–16·3	38	18·3	17·7–19·1	3	21·1	20·1–23·8	17
Carbohydrate (g)	–	194·1	167·2–233·8	–	230·1	193·1–266·9		192·5	170·7–226·3		173·6	144·8–198·9	
Carbohydrate (%E)-AMDR	45–65	39·6	35·9–43·5	85	42·3	39·0–46·7	72	40·4	39·2–43·8	87	35·7	32·5–39·6	97
Total fat (g)	–	77·3	64·8–93·8	–	87·4	75·5–104·1	–	74·5	62·6–85·0	–	72·9	61·9–93·1	–
Total fat (%E)- AMDR	20–35	35·9	31·3–38·7	55	36·6	30·9–38·5	62	35·9	32·4–39·1	57	34·5	30·0–38·8	45
Saturated fat (g)	–	28·5	23·4–36·5	–	34·9	26·8–41·1	–	27·8	23·6–36·5	–	25·4	20·8–30·4	–
Saturated fat (%E)-AMDR	< 10	12·9	11·4–15·5	92	14·3	12·2–16·0	93	13·5	11·5–16·0	97	11·6	10·6–12·9	86
Linoleic acid (g)- AI	F: 8 M: 13	9·9	7·7–12·3	47	10·6	8·3–13·9	45	8·9	7·1–11·1	57	10·1	8·1–12·2	38
Linoleic acid (%E)-AMDR	4–10	4·4	3·8–5·4	39	4·3	3·7–5·4	35	4·5	3·6–5·2	50	4·7	3·9–5·7	31
Alpha-linolenic acid (g)-AI	F: 0·8 M: 1·3	1·5	1·2–2·0	14	1·7	1·2–2·3	21	1·5	1·1–1·9	13	1·4	1·2–1·9	7
Alpha-linolenic acid (%E)-AMDR	0·4–1·0	0·7	0·5–0·9	27	0·7	0·5–1·0	35	0·7	0·5–0·9	27	0·7	0·5–0·9	21
Long-chain *n*-3 (mg)-AI	F: 90 M: 160	338·3	125·5–914·5	18	213·0	93·4–730·3	35	333·4	136·0–1068·4	13	452·6	231·1–868·9	7
Dietary fibre (g)-AI	F: 25 M: 30	25·6	21·5–30·3	52	27·2	23·6–33·2	38	24·6	20·5–28·7	60	24·4	21·4–29·9	59
Dietary fibre (%E)	–	2·5	2·1–2·9	–	2·4	2·0–3·0	–	2·5	2·0–2·8	–	2·6	2·3–3·1	–
Free sugar (g)	50	23·2	14·5–38·5	13	42·6	34·7–59·3	34	23·2	16·4–31·6	3	14·0	8·2–17·7	0
Free sugar (%E)	< 10	4·7	2·9–7·7	10	9·0	6·7–10·2	27	4·8	3·2–7·1	3	3·1	1·7–3·8	0
Alcohol (std drink)	≤ 1·43	0·2	0·0–1·4	25	0·2	0·0–1·4	24	0·1	0·0–0·8	20	0·3	0·0–1·8	31
Alcohol (%E)	–	1·0	0·0–4·2	–	0·7	0·0–4·4	–	0·6	0·0–2·9	–	1·0	0·0–6·6	–
Thiamin (mg)-EAR	F: 0·9 M: 1·0	1·4	1·2–1·7	5	1·44	1·1–1·8	10	1·3	1·1–1·6	0	1·5	1·2–1·7	3
Riboflavin (mg)-EAR	F: 51–70 yo: 0·9 71 yo+: 1·1 M: 51–70 yo: 1·1 71 yo+: 1·3	1·8	1·4–2·2	3	1·8	1·5–2·2	7	1·7	1·4–2·2	0	1·7	1·4–2·4	3
Vitamin C-EAR	30	92·5	62·1–115·4	5	92·3	77·4–113·0	3	96·8	52·1–111·3	10	83·3	57·1–153·9	0
Vitamin E (mg)-AI	F: 7 M: 10	11·8	9·8–15·0	14	13·4	9·7–16·5	24	10·7	8·7–13·8	7	11·9	10·3–14·5	10
Vitamin B_6_ (mg)-EAR	F: 1·3 M: 1·4	1·4	1·1–1·7	46	1·4	1·0–1·6	52	1·2	1·1–1·8	57	1·4	1·3–1·7	28
Folate (ug)-EAR	320	660·7	520·8–785·7	1	616·7	466·2–804·0	3	665·6	542·2–791·2	0	688·3	527·5–754·8	0
Vitamin B_12_ (ug)-EAR	2·0	4·2	3·3–5·3	2	3·7	3·0–4·9	3	4·3	3·5–5·2	0	4·3	3·7–5·8	3
Ca (mg)-EAR	F: 1100 M: 51–70 yo: 840 71 yo+: 1100	829·9	681·6–1047·7	71	843·9	687·9–1095·9	72	848·3	684·5–1018·4	70	773·1	607·2–1135·8	69
Iodine (ug)- EAR	100	174·5	149·5–213·1	3	175·0	151·4–202·9	7	182·0	153·9–224·2	0	170·5	135·8–201·3	3
Fe (mg)-EAR	F: 5 M: 6	10·9	9·5–13·4	0	11·8	9·8–14·0	0	10·3	8·6–13·0	0	10·7	9·8–13·2	0
Zn (mg)-EAR	F: 6·5 M: 12	10·1	8·9–12·4	21	10·3	8·7–11·9	31	9·5	8·6–11·2	17	10·6	9·1–13·1	14
Potassium (mg)- AI	F: 2800 M: 3800	3135·2	2661·4–3646·1	51	3135·0	2743·9–3601·1	55	2861·8	2490·8–3594·6	57	3205·0	2753·7–3911·5	41
Na (mg)-AI to SDT	460–2000	2130·1	1699·2–2683·0	55	2134·4	1697·2–2800·1	55	1978·4	1688·8–2582·9	50	2130·8	1709·8–2620·4	59
Food groups and food subgroups													
Vegetables	F: 5 M: 51–70 yo: 5·5 71 yo+: 5·0	3·2	2·3–4·2	82	3·6	2·7–5·1	79	2·7	1·9–3·6	93	3·2	2·4–5·3	72
Dark green	–	0·2	0·1–0·3	–	0·2	0·1–0·3	–	0·1	0·0–0·2	–	0·2	0·1–0·3	–
Red and orange	–	0·6	0·3–1·0	–	0·6	0·4–1·0	–	0·5	0·3–1·0	–	0·6	0·3–1·1	–
Legumes	–	0·0	0·0–0·2	–	0·0	0·0–0·2	–	0·0	0·0–0·3	–	0·0	0·0–0·2	–
Other	–	1·2	0·8–1·7	–	1·3	1·0–2·3	–	0·9	0·5–1·5	–	1·2	0·9–2·3	–
Starchy	–	0·8	0·4–1·4	–	0·8	0·4–1·8	–	0·6	0·3–1·4	–	0·9	0·4–1·1	–
Starchy (%)	–	27·3	14·1–42·2	–	29·7	13·4–48·8	–	27·7	11·4–42·9	–	25·0	14·2–36·2	–
Meat and alternatives	2	2·3	1·8–3·0	44	1·9	1·7–2·4	72	2·2	1·8–2·9	47	3·0	2·6–3·5	14
Red meat	≤ 1	0·5	0·3–0·8	11	0·3	0·1–0·6	7	0·5	0·3–0·7	7	0·8	0·6–1·0	21
Processed meat	–	0·1	0·0–0·3	–	0·1	0·0–0·3		0·1	0·0–0·3		0·1	0·0–0·4	
Seafood	≥ 0·3	0·3	0·1–0·6	50	0·2	0·0–0·5	66	0·3	0·1–0·6	50	0·4	0·1–0·6	35
Nuts and seeds	–	0·5	0·1–0·9	–	0·4	0·1–1·1	–	0·4	0·3–0·9	–	0·5	0·3–0·7	–
Legumes	–	0·0	0·0–0·1	–	0·0	0·0–0·1	–	0·0	0·0–0·2	–	0·0	0·0–0·1	–
Soya products	–	0·0	0·0–0·0	–	0·0	0·0–0·0	–	0·0	0·0–0·0	–	0·0	0·0–0·0	–
Poultry	–	0·3	0·0–0·7	–	0·2	0·0–0·7	–	0·2	0·0–0·5	–	0·6	0·3–0·8	–
Eggs	–	0·3	0·2–0·4	–	0·2	0·1–0·3	–	0·3	0·2–0·4	–	0·3	0·2–0·4	–
Dairy and alternatives	F: 4 M: 51–70 yo: 2·5 71 yo+: 3·5	1·6	1·2–2·3	89	1·6	1·3–2·3	90	1·7	1·2–2·4	87	1·4	1·1–2·6	90
Milk	–	0·9	0·5–1·5	–	1·0	0·5–1·4	–	1·0	0·7–1·7	–	0·7	0·3–1·4	–
Cheese	–	0·3	0·1–0·6	–	0·3	0·1–0·5	–	0·3	0·0–0·4	–	0·3	0·2–0·7	–
Yoghurt	–	0·1	0·0–0·3	–	0·1	0·0–0·4	–	0·1	0·0–0·3	–	0·1	0·0–0·4	–
Milk alternatives	–	0·0	0·0–0·0	–	0·0	0·0–0·0	–	0·0	0·0–0·0	–	0·0	0·0–0·0	–
Fruits	2	1·4	0·9–1·9	77	1·5	0·9–1·8	83	1·4	0·7–2·0	73	1·4	1·1–2·1	76
Citrus, melons and berries	–	0·2	0·1–0·5	–	0·2	0·0–0·5	–	0·2	0·1–0·6	–	0·2	0·1–0·4	–
Other fruit	–	1·0	0·5–1·5	–	0·9	0·5–1·4	–	0·9	0·4–1·6	–	1·1	0·7–1·5	–
Fruit juice	–	0·0	0·0–0·0	–	0·0	0·0–0·2	–	0·0	0·0–0·0	–	0·0	0·0–0·0	–
Fruit juice (%)	–	0·0	0·0–0·5	–	0·0	0·0–10·4	–	0·0	0·0–0·0	–	0·0	0·0–0·0	–
Grains	F: 51–70 yo: 4 71 yo+: 3 M: 51–70 yo: 6 71 yo+: 4·5	5·5	4·3–6·6	23	5·8	4·5–7·3	21	5·6	4·6–6·8	17	5·0	4·0–6·3	31
Refined grains	–	3·2	2·2–4·1	–	3·3	2·3–4·4	–	3·2	2·4–4·1	–	3·1	1·9–3·9	–
Wholegrains	–	2·2	1·3–3·5	–	1·9	1·1–3·7	–	2·5	1·3–3·7	–	2·1	1·2–2·7	–
Wholegrains (%)	≥ 67	43·4	28·7–56·1	89	40·6	23·2–53·8	90	44·4	29·2–59·8	87	44·3	28·8–58·5	90
Discretionary Foods*	F: 51–70 yo: 0–2·5 71 yo+: 0–2 M: 0–2·5	16·1	12·0–21·2	100	21·5	17·8–27·6	100	15·5	12·1–19·6	100	12·0	10·0–15·5	100

P:NP, protein to non-protein; IQR, interquartile range; kJ, kilojoule; BW, body weight; EAR, estimated average requirement; F, female; yo, years old; M, male; %E, as a percentage of energy; AMDR, acceptable macronutrient distribution range; AI, adequate intake; std, standard; SDT, suggested dietary target.

* (1 discretionary food serve = 4·8 g solid fat equivalents, 4·2 g added sugars or 10 g alcohol).

A large proportion of participants had intakes below the recommendations for vegetables (82 %), fruits (77 %), dairy and alternatives (89 %), wholegrain % (89 %), meat and alternatives (44 %), seafood (50 %) and nutrients found in those food groups including dietary fibre (52 %), vitamin B_6_ (46 %), Ca (71 %) and K (51 %), while on the contrary exceeded the recommendations for discretionary foods (100 %) and related nutrients including saturated fat %E (92 %) and Na (55 %). Only a small proportion had intakes below the recommendations for protein per kg body weight (1 %), folate (1 %) and vitamin B_12_ (2 %). No participants had intakes below recommendations for Fe.

### Associations between dietary P:NP and intakes of dietary components

Table 3 presents the adjusted analyses on the associations between dietary P:NP and intakes of energy, nutrients, food groups and food subgroups (for unadjusted analyses see Supplementary Table S4, Online Resource). As a continuous variable, dietary P:NP (per 1 % increment) was associated with lower intakes of energy (*β* –80·95 (95 % CI: –154·42, –7·48, *P* = 0·031)), discretionary foods (*β* –0·89 (95 % CI: –1·17, –0·61, *P* < 0·001)) and related nutrients including carbohydrate (*β* –3·86 (95 % CI: –5·90, –1·81, *P* < 0·001)), carbohydrate %E (*β* –0·42 (95 % CI: –0·72, –0·12, *P* = 0·007)), total fat (*β* –1·29 (95 % CI: –2·32, –0·26, *P* = 0·015)), saturated fat (*β* –0·76 (95 % CI: –1·18, –0·34, *P* < 0·001)), saturated fat %E (*β* –0·21 (95 % CI: –0·34, –0·08, *P* = 0·002)), free sugar (*β* –2·64 (95 % CI: –3·55, –1·73, *P* < 0·001)) and free sugar %E (*β* –0·50 [95 % CI: –0·69, –0·31, *P* < 0·001)) in adjusted analyses. Dietary P:NP (per 1 % increment) was also associated with higher intakes of meat and alternatives (*β* 0·09 (95 % CI: 0·05, 0·12, *P* < 0·001)), red meat (*β* 0·05 (95 % CI: 0·03, 0·07, *P* < 0·001), poultry (*β* 0·03 (95 % CI: 0·01, 0·05, *P* = 0·004)) and wholegrain % (*β* 1·17 (95 % CI: 0·10, 2·23, *P* = 0·032)) and nutrients found in those food groups including protein (*β* 1·97 (95 % CI: 1·19, 2·75, *P* < 0·001)), protein %E (*β* 0·60 (95 % CI: 0·55, 0·64, *P* < 0·001)), vitamin B_12_ (*β* 0·11 (95 % CI: 0·02, 0·19, *P* = 0·013)) and Zn (*β* 0·16 (95 % CI: 0·02, 0·31, *P* = 0·023)) in adjusted analyses.

**Table 3:** Associations between dietary protein to non-protein ratios with intakes of energy, nutrients, food groups and food subgroups in adjusted analyses, using linear regression presented as beta coefficients

Dietary component	Low P:NP 0·19 (0·18, 0·21)		Medium P:NP 0·24 (0·23, 0·25)		High P:NP 0·29 (0·28, 0·33)		As continuous variable P:NP % (each 1 % increment)	
	*β*	95 % CI	*β*	95 % CI	*β*	95 % CI	*β*	95 % CI
Energy (kJ)	Ref		−812·35	–1660·22, 35·53	−875·61	–1844·11, 92·90	−80·95	–154·42, –7·48
Nutrients								
Protein (g/kg BW)	Ref		0·09	–0·04, 0·22	0·35	0·20, 0·50	0·03	0·02, 0·04
Protein (g)	Ref		5·10	–4·34, 14·54	22·28	11·49, 33·06	1·97	1·19, 2·75
Protein (%E)	Ref		2·69	1·63, 3·74	6·71	5·50, 7·91	0·60	0·55, 0·64
Carbohydrate (g)	Ref		−29·14	–52·17, –6·12	−53·79	–80·10, –27·49	−3·86	–5·90, –1·82
Carbohydrate (%E)	Ref		−1·79	–5·06, 1·48	−7·03	–10·77, –3·30	−0·42	–0·72, –0·12
Total fat (g)	Ref		−13·80	–25·75, –1·86	−10·90	–24·54, 2·75	−1·29	–2·32, –0·26
Total fat (%E)	Ref		−2·48	–5·86, 0·90	−1·01	–4·87, 2·86	−0·21	–0·50, 0·08
Saturated fat (g)	Ref		−3·53	–8·62, 1·57	−7·28	–13·10, –1·47	−0·76	–1·18, –0·34
Saturated fat (%E)	Ref		−0·24	–1·81, 1·33	−1·93	–3·72, –0·13	−0·21	–0·34, –0·08
Linoleic acid (g)	Ref		−2·48	–4·47, –0·49	0·19	–2·09, 2·46	−0·05	–0·02, 0·01
Linoleic acid (%E)	Ref		−0·01	–0·02, 0·00	0·01	0·00, 0·02	0·00	0·00, 0·00
Alpha–linolenic acid (g)	Ref		−0·57	–1·13, 0·00	−0·48	–1·12, 0·17	−0·04	–0·09, 0·01
Alpha-linolenic acid (%E)	Ref		0·00	0·00, 0·00	0·00	0·00, 0·00	0·00	0·00, 0·00
Long-chain *n*-3 (mg)	Ref		184·31	–118·12 486·74	185·91	–159·56 531·37	16·26	–10·03, 42·55
Dietary fibre (g)	Ref		−3·28	–8·50, 1·94	0·20	–5·76, 6·17	−0·08	–0·54, 0·38
Dietary fibre (%E)	Ref		−0·33	–0·90, 0·25	0·12	–0·54, 0·78	0·00	–0·05, 0·05
Free sugar (g)	Ref		−23·19	–32·71, –13·67	−38·09	–48·97, –27·21	−2·64	–3·55, –1·73
Free sugar (%E)	Ref		−4·05	–6·04, –2·05	−7·38	–9·66, –5·11	−0·50	–0·69, –0·31
Alcohol (std drink)	Ref		0·44	–0·25, 1·12	0·09	–0·69, 0·87	−0·01	–0·07, 0·05
Alcohol (%E)	Ref		1·70	–0·75, 4·14	0·85	–1·95, 3·64	0·00	–0·22, 0·21
Thiamin (mg)	Ref		−0·10	–0·54, 0·34	0·04	–0·46, 0·55	0·02	–0·02, 0·05
Riboflavin (mg)	Ref		0·11	–0·33, 0·56	0·06	–0·45, 0·57	0·02	–0·02, 0·06
Vitamin C (mg)	Ref		−18·03	–54·56, 18·51	1·29	–40·45, 43·02	−0·09	–3·30, 3·11
Vitamin E (mg)	Ref		−3·42	–6·74, –0·09	−0·61	–4·41, 3·19	−0·12	–0·42, 0·18
Vitamin B_6_ (mg)	Ref		0·03	–0·26, 0·32	0·30	–0·03, 0·63	0·02	0·00, 0·05
Folate (ug)	Ref		42·86	–98·48 184·20	5·25	–156·20 166·69	−0·56	–12·86, 11·74
Vitamin B12 (ug)	Ref		0·98	–0·01, 1·96	1·29	0·17, 2·42	0·11	0·02, 0·19
Ca (mg)	Ref		37·66	–186·34 261·66	9·54	–246·34 265·41	1·09	–18·34, 20·53
I (ug)	Ref		36·17	–7·99, 80·34	17·10	–33·35, 67·54	1·05	–2·87, 4·96
Fe (mg)	Ref		0·16	–1·66, 1·98	0·62	–1·46, 2·70	0·06	–0·10, 0·22
Zn (mg)	Ref		0·85	–0·78, 2·49	2·07	0·21, 3·94	0·16	0·02, 0·31
K (mg)	Ref		−91·98	–652·48 468·53	294·45	–345·80 934·70	22·76	–26·14, 71·67
Na (mg)	Ref		20·25	–395·82 436·32	−82·70	–557·97 392·57	−17·31	–53·14, 18·53
Food groups and food subgroups								
Vegetables	Ref		−1·49	–2·88, –0·09	−0·37	–1·96, 1·23	−0·06	–0·19, 0·06
Dark green	Ref		−0·05	–0·22, 0·13	0·00	–0·20, 0·20	0·00	–0·02, 0·01
Red and orange	Ref		−0·08	–0·52, 0·37	0·06	–0·45, 0·56	0·00	–0·04, 0·04
Legumes	Ref		0·02	–0·11, 0·14	0·06	–0·09, 0·20	0·00	–0·01, 0·01
Other	Ref		−1·03	–1·95, –0·10	−0·17	–1·23, 0·89	−0·03	–0·11, 0·06
Starchy	Ref		−0·36	–0·88, 0·17	−0·30	–0·90, 0·30	−0·03	–0·07, 0·02
Starchy (%)	Ref		−0·51	–12·48, 11·46	−3·30	–16·97, 10·38	−0·42	–1·45, 0·62
Meat and alternatives	Ref		0·18	–0·22, 0·58	1·06	0·61, 1·52	0·09	0·05, 0·12
Red meat	Ref		0·23	0·02, 0·43	0·64	0·41, 0·87	0·05	0·03, 0·07
Processed meat	Ref		0·05	–0·08, 0·19	0·07	–0·08, 0·23	0·00	–0·01, 0·01
Seafood	Ref		0·07	–0·10, 0·24	0·14	–0·05, 0·34	0·01	0·00, 0·03
Nuts and seeds	Ref		−0·08	–0·38, 0·22	−0·02	–0·36, 0·32	−0·01	–0·03, 0·02
Legumes	Ref		0·02	–0·05, 0·09	0·02	–0·06, 0·10	0·00	–0·01, 0·01
Soya products	Ref		−0·02	–0·07, 0·03	−0·04	–0·09, 0·02	0·00	–0·01 0·00
Poultry	Ref		−0·16	–0·38, 0·06	0·17	–0·08, 0·42	0·03	0·01, 0·05
Eggs	Ref		0·09	–0·03, 0·20	0·06	–0·08, 0·19	0·00	–0·01, 0·00
Dairy and alternatives	Ref		0·34	–0·32, 0·99	0·09	–0·65, 0·84	0·01	–0·04, 0·07
Milk	Ref		0·57	–0·05, 1·18	−0·01	–0·71, 0·69	0·00	–0·05, 0·06
Cheese	Ref		−0·08	–0·34, 0·19	−0·01	–0·31, 0·29	−0·01	–0·03, 0·02
Yoghurt	Ref		−0·15	–0·35, 0·04	−0·05	–0·27, 0·17	0·01	–0·01, 0·02
Milk alternatives	Ref		0·00	–0·12, 0·12	0·16	0·03, 0·30	0·01	0·00, 0·02
Fruits	Ref		−0·41	–0·91, 0·10	−0·12	–0·69, 0·46	0·01	–0·03, 0·06
Citrus, melons and berries	Ref		0·06	–0·16, 0·28	0·01	–0·24, 0·26	0·00	–0·02, 0·02
Other fruit	Ref		−0·32	–0·73, 0·08	0·03	–0·43, 0·49	0·01	–0·02, 0·05
Fruit juice	Ref		−0·14	–0·31, 0·03	−0·16	–0·35, 0·04	0·00	–0·02, 0·01
Fruit juice (%)	Ref		−5·90	–13·85, 2·06	−9·82	–18·91, –0·74	−0·35	–1·07, 0·36
Grains	Ref		0·24	–0·76, 1·25	−0·10	–1·25, 1·04	−0·02	–0·11, 0·07
Refined grains	Ref		−0·34	–1·21, 0·54	−0·49	–1·48, 0·51	−0·07	–0·14, 0·01
Wholegrains	Ref		0·58	–0·30, 1·46	0·38	–0·63, 1·39	0·05	–0·03, 0·12
Wholegrains (%)	Ref		10·78	–1·54, 23·10	12·33	–1·75, 26·40	1·17	0·10, 2·23
Discretionary foods*	Ref		−6·38	–9·57, –3·19	−11·44	–15·08, –7·79	−0·89	–1·17, –0·61

P:NP, protein to non-protein; kJ, kilojoule; BW, body weight; %E, as a percentage of energy.

Adjusted by socio-demographic, lifestyle factors and health (age, sex, BMI, country of birth, marital status, education level, source of income, housing arrangement, living location, smoking status, PASE, ability to prepare own meals and self-rated health) (*n* 71).

* (1 discretionary food serve = 4·8 g solid fat equivalents, 4·2 g added sugars or 10 g alcohol).

Compared with the low dietary P:NP tertile, both the medium and high tertiles were associated with lower intakes of discretionary foods (medium tertile: *β* –6·38 (95 % CI: –9·57, –3·19, *P* < 0·001) and high tertile *β* –11·44 (95 % CI: –15·08, –7·79, *P* < 0·001)) and related nutrients including carbohydrate (medium tertile: *β* –29·14 (95 % CI: –52·17, –6·12, *P* = 0·014) and high tertile *β* –53·79 (95 % CI: –80·10, –27·49, *P* < 0·001)), free sugar (medium tertile: *β* –23·19 (95 % CI: –32·71, –13·67, *P* < 0·001) and high tertile *β* –38·09 (95 % CI: –48·97, –27·21, *P* < 0·001)) and free sugar %E (medium tertile: *β* –4·05 (95 % CI: –6·04, –2·05, *P* < 0·001) and high tertile *β* –7·38 (95 % CI: –9·66, –5·11, *P* < 0·001)) in adjusted analyses. Both the medium and high dietary P:NP tertiles were also associated with higher intakes of red meat (medium tertile: *β* 0·23 (95 % CI: 0·02, 0·43, *P* = 0·031) and high tertile *β* 0·64 (95 % CI: 0·41, 0·87, *P* < 0·001)) in adjusted analyses.

Compared with the low dietary P:NP tertile, the medium tertile was associated with lower intakes of vegetables (*β* –1·49 (95 % CI: –2·88, –0·09, *P* = 0·037)) and other vegetables (*β* –1·03 (95 % CI: –1·95, –0·10, *P* = 0·031)), total fat (*β* –13·80 (95 % CI: –25·75, –1·86, *P* = 0·024)), linoleic acid (*β* –2·48 (95 % CI: –4·47, –0·49, *P* = 0·016)) and vitamin E (*β* –3·42 (95 % CI: –6·74, –0·09, *P* = 0·044)) in adjusted analyses.

Compared with the low dietary P:NP tertile, the high tertile was associated with lower intakes of fruit juice % (*β* –9·82 (95 % CI: –18·91, –0·74, *P* = 0·035)), carbohydrate %E (*β* –7·03 (95 % CI: –10·77, –3·30, *P* < 0·001)), saturated fat (*β* –7·28 (95 % CI: –13·10, –1·47, *P* = 0·015)) and saturated fat %E (*β* –1·93 (95 % CI: –3·72, –0·13, *P* = 0·036)) and higher intakes of meat and alternatives (*β* 1·06 (95 % CI: 0·61, 1·52, *P* < 0·001)), milk alternatives (*β* 0·16 (95 % CI: 0·03, 0·30, *P* = 0·020)) and related nutrients including vitamin B_12_ (*β* 1·29 (95 % CI: 0·17, 2·42, *P* = 0·025)) and Zn (*β* 2·07 (95 % CI: 0·21, 3·94, *P* = 0·030)) in adjusted analyses.

## Discussion

To our knowledge, this is the first study to examine the associations between dietary P:NP and intakes of other dietary components in older adults. Higher dietary P:NP was associated with lower intakes of energy, nutrients and a food group known to increase risk of chronic disease (saturated fat, saturated fat %E, free sugar, free sugar %E and discretionary foods) and higher intake of a dietary component known to reduce risk of chronic disease (wholegrain %)^(11–13)^. Unsurprisingly, higher dietary P:NP was associated with a higher consumption of nutrients found in protein-rich foods and a protein-rich food group, which are also known to be at risk of deficiency in ageing (vitamin B_12_, Zn and meat and alternatives), and a protein-rich food subgroup associated with increased risks of chronic disease (red meat)^(7,11,12)^. Majority of participants consumed habitual diets that were nutritionally inadequate: many had intakes below recommendations for vegetables, fruits, dairy and alternatives, wholegrain %, meat and alternatives, seafood, dietary fibre, vitamin B_6_, Ca and K, but exceeded the recommendations for discretionary foods, saturated fat %E and Na. Compared with carbohydrate and fat, most participants had protein intakes within recommendations. We found that almost all participants consumed absolute protein intakes within recommendations. Furthermore, we observed that there was less variation in protein %E than carbohydrate %E and fat %E intakes, which is consistent with previous research demonstrating Protein Leverage^(1,34)^.

### Nutritional adequacy

The substantial proportions of inadequate nutritional intakes in this cohort were surprising yet consistent with studies involving nutrition intervention participants interested in nutrition^(35,36)^. Majority had intakes below recommendations for vegetables, fruits, dairy and alternatives, wholegrain %, meat and alternatives and seafood, and thus also had intakes below recommendations for nutrients found in these food groups including dietary fibre, vitamin B_6_, Ca and K. These food groups and nutrients are important for the prevention of chronic diseases such as CVD, diabetes and cancer^(12,13)^. Simultaneously, most exceeded the recommendations for discretionary foods, and thus also exceeded recommendations for saturated fat %E and Na, that are associated with increased risks of chronic disease^(11,12)^.

On the bright side, previous research has found the prevalence of nutritional inadequacies to be lower amongst older adults participating in nutrition intervention studies than other groups including those of the highest social class^(37)^. The proportions of nutritional inadequacy in the current study were lower for thiamin, riboflavin, vitamin C, vitamin E, folate, vitamin B_12_, iodine and Fe than those found in a systematic review and meta-analysis on micronutrient intakes in older adults aged 65+ years from thirty-seven observational studies, not based on nutrition intervention^(38)^. Participants’ intakes of most food groups and food subgroups were also better matched to recommendations than the general population: the proportions below recommendations were lower for vegetables, dairy and alternatives, meat and alternatives and grains, whilst red meat intake was also lower and did not exceed recommendations compared with adults aged 51+ years in the Australian National Nutrition and Physical Activity Survey 2011–2012^(39)^. However, the proportion below recommendations for fruits was higher, and there were lower intakes of fruits, seafood and wholegrain % and higher discretionary intake in the current study^(40,41)^. The proportions below recommendations were also lower for vegetables and higher for fruits compared with adults aged 65–74 years in the Australian National Health Survey 2017–2018^(41)^.

Although majority had intakes below the recommendations for carbohydrate %E and more than half exceeded the recommendations for total fat %E, most consumed protein %E intake within recommendations. Thus, the median intakes of protein %E were similar, and carbohydrate %E was lower, whilst total fat %E including saturated fat %E was higher than adults aged 51+ years in the Australian National Nutrition and Physical Activity Survey 2011–2012^(40)^. The macronutrient distribution consumed in the current study was also comparable to adults aged 51+ years in the Australian National Nutrition and Physical Activity Survey 2011–2012 in the ratio of one part protein to four parts non-protein (fat and carbohydrate), or one part protein to two parts carbohydrate^(40)^. Research using model life tables and national macronutrient supplies for a subset of developed countries found that overnutrition from increasing total energy, carbohydrate %E and fat %E supplies increased mortality^(42)^. Furthermore, emerging research showed that macronutrient requirements may change across the lifespan and diets with equal parts protein to carbohydrate^(43)^ and moderate to high protein %E intakes^(44)^ reduced mortality in later life.

It is notable that we observed such high rates of nutritional inadequacy even though participants in the NHL study were of high socio-economic background and rated their health as excellent/good. There are a number of factors influencing nutritional intakes that go beyond self-perceived health and income. For instance, factors such as social support, taste perception, health beliefs, willingness to change, nutrition knowledge, awareness of dietary guidelines and health systems incorporating nutrition care can all affect dietary intake^(45,46)^. Nutrition education programs can motivate older adults to improve nutritional intakes through changing nutrition knowledge, attitude and behaviours^(46)^, as it was found that nutrition awareness and use of nutrition information were associated with adherence to dietary guidelines^(47,48)^. Future research should investigate strategies to address other determinants of nutritional intake in older adults.

### Associations between dietary P:NP and intakes of dietary components

Dietary P:NP was inversely associated with intakes of energy, saturated fat, saturated fat %E, free sugar, free sugar %E and discretionary foods. Similar to the present study, it has been observed in the American population in analyses of National Health and Nutrition Examination Survey data that protein %E intake was inversely associated with ultra-processed food %E intake, whilst carbohydrate %E, added sugar %E, saturated fat %E and total energy intakes were positively associated with ultra-processed food %E intakes^(2,5)^. The Protein Leverage Hypothesis states that absolute protein intakes are regulated more strongly than fat and carbohydrate, resulting in excess energy consumption and predisposing to overweight and obesity on protein-dilute diets^(1)^. Hence, the protein diluting effects of discretionary foods could explain the inverse associations between dietary P:NP and energy intake.

Dietary P:NP was positively associated with intakes of vitamin B_12_, Zn, meat and alternatives, red meat, poultry and wholegrain %. The protein source, such as from plant or animal foods, could have different impacts on the associations of dietary P:NP with intakes of other dietary components. Future research should consider whether the associations between dietary P:NP and intakes of other dietary components are modified on semi-vegetarian and plant-based diets.

There are some study limitations. First, causation could not be assessed due to the cross-sectional design. Second, although the sample size was powered for the NHL study, the sample size was small for the current study. Third, Na and free sugar intake was likely underestimated; Na added in home prepared foods or at the table^(49)^ and sugars from honey and fruit juice^(18)^ were not accounted for, respectively. Fourth, the number serves of food groups and food subgroups were obtained using FoodWorks 8, which is based on the Australian Guide to Healthy Eating, but there are discrepancies on serve size equivalents, and we used an adapted definition for a serve of discretionary. Additionally, dietary P:NP was used to demonstrate the impact Protein Leverage could have on the intakes of other dietary components, and thus did not differentiate between the different types of carbohydrate and fat such as refined or unrefined carbohydrates and saturated or unsaturated fats. Lastly, participants were volunteers for a nutrition intervention study and majority were female, reflecting a highly motivated group and an imbalance in male representation. There were also strengths. Dietary assessment was conducted using a prospective seven-day weighed food record which is often used as a reference method. Sensitivity analyses were conducted and non-plausible reporters were excluded to better reflect true intakes. Furthermore, we accounted for confounders including socio-demographic, lifestyle and health factors in analyses between dietary P:NP and intakes of other dietary components.

In conclusion, we showed that diets with higher dietary P:NP were associated with lower intakes of energy, saturated fat, free sugar and discretionary foods and higher intakes of vitamin B_12_, Zn, meat and alternatives, red meat, poultry and wholegrain %. Vice versa for diets with lower dietary P:NP, indicating the potential protein diluting or protein-enriching effects of these dietary components. The current study also demonstrated that the majority of individuals aged 65–75 years from a high socio-economic background had inadequate nutritional intakes.

## Supplementary material

For supplementary material accompanying this paper visit https://doi.org/10.1017/S1368980023001726
